# Multipanel Approach including miRNAs, Inflammatory Markers, and Depressive Symptoms for Metabolic Dysfunction-Associated Steatotic Liver Disease Diagnosis during 2-Year Nutritional Intervention

**DOI:** 10.3390/nu16111547

**Published:** 2024-05-21

**Authors:** Ana Luz Tobaruela-Resola, José I. Riezu-Boj, Fermin I. Milagro, Paola Mogna-Pelaez, José I. Herrero, Mariana Elorz, Alberto Benito-Boillos, Josep A. Tur, J. Alfredo Martínez, Itziar Abete, M. Angeles Zulet

**Affiliations:** 1 Department of Nutrition, Food Sciences and Physiology and Centre for Nutrition Research, Faculty of Pharmacy and Nutrition, University of Navarra, 31008 Pamplona, Spain; atobaruela@unav.es (A.L.T.-R.); jiriezu@unav.es (J.I.R.-B.); fmilagro@unav.es (F.I.M.); pmogna@alumni.unav.es (P.M.-P.); iabetego@unav.es (I.A.); 2 Navarra Institute for Health Research (IdiSNA), 31008 Pamplona, Spain; iherrero@unav.es (J.I.H.); marelorz@unav.es (M.E.); albenitob@unav.es (A.B.-B.); 3 Biomedical Research Centre Network in Physiopathology of Obesity and Nutrition (CIBERobn), Instituto de Salud Carlos III, 28029 Madrid, Spain; pep.tur@uib.es (J.A.T.); jalfredo.martinez@imdea.org (J.A.M.); 4 Liver Unit, Clínica Universidad de Navarra, 31008 Pamplona, Spain; 5 Biomedical Research Centre Network in Hepatic and Digestive Diseases (CIBERehd), 28029 Madrid, Spain; 6 Department of Radiology, Clínica Universidad de Navarra, 31008 Pamplona, Spain; 7 Research Group on Community Nutrition and Oxidative Stress, University of Balearic Islands-IUNICS & IDISBA, 07122 Palma, Spain; 8 Precision Nutrition and Cardiovascular Health Program, IMDEA Food, CEI UAM + CSIC, 28049 Madrid, Spain

**Keywords:** MASLD, biomarkers, circulating miRNA, inflammatory status, NAFLD, weight loss, dietary intervention, depression, fatty liver, long-term follow-up

## Abstract

Metabolic dysfunction-associated steatotic liver disease (MASLD), with a prevalence of 30% of adults globally, is considered a multifactorial disease. There is a lack of effective non-invasive methods for accurate diagnosis and monitoring. Therefore, this study aimed to explore associations between changes in circulating miRNA levels, inflammatory markers, and depressive symptoms with hepatic variables in MASLD subjects and their combined potential to predict the disease after following a dietary intervention. Biochemical markers, body composition, circulating miRNAs and hepatic and psychological status of 55 subjects with MASLD with obesity and overweight from the FLiO study were evaluated by undergoing a 6-, 12- and 24-month nutritional intervention. The highest accuracy values of combined panels to predict the disease were identified after 24 months. A combination panel that included changes in liver stiffness, high-density lipoprotein cholesterol (HDL-c), body mass index (BMI), depressive symptoms, and triglycerides (TG) yielded an AUC of 0.90. Another panel that included changes in hepatic fat content, total cholesterol (TC), miR15b-3p, TG, and depressive symptoms revealed an AUC of 0.89. These findings identify non-invasive biomarker panels including circulating miRNAs, inflammatory markers, depressive symptoms and other metabolic variables for predicting MASLD presence and emphasize the importance of precision nutrition in MASLD management and the sustained adherence to healthy lifestyle patterns.

## 1. Introduction

Metabolic dysfunction-associated steatotic liver disease (MASLD) is a leading cause of chronic liver disease, affecting 30% of the global adult population. Its prevalence has increased, paralleling the increased prevalence of obesity-related diseases [1].

MASLD encompasses a spectrum of liver conditions varying in severity. Metabolic dysfunction-associated steatohepatitis (MASH) is characterized by a more severe process involving inflammation and hepatocyte damage; typically, MASH is accompanied by pericellular fibrosis, which may progress to cirrhosis and liver cancer [2]. Linked with obesity, type 2 diabetes mellitus (T2DM), dyslipidemia, and hypertension, MASLD yields extra-hepatic complications, including cardiovascular and kidney diseases, vascular dysfunction, arterial remodeling, and cognitive impairment [3].

MASLD is characterized as a multisystem disorder, with various factors contributing to the interindividual differences observed in the development and progression [4], such as genetic and environmental factors [5] as well as alterations in the communication between various organs and tissues (adipose tissue, pancreas, gut, and liver) [6]. Epigenetic mechanisms, particularly microRNA (miRNA) dysregulation, significantly influence metabolic disorders associated with MASLD. MiRNAs, which are small non-coding RNAs, have a significant impact on MASLD and its progression to more severe stages [7]. The progression of the disease is also significantly influenced by oxidative stress, lipotoxicity, and inflammation [8]. Pro-inflammatory cytokines produced by visceral adipose tissue (VAT) in individuals with metabolic dysfunction contribute to the onset of insulin resistance (IR), hepatic lipotoxicity, and MASLD [9]. Chronic systemic inflammation, hyperammonemia, genetic predisposition, and intestinal dysbiosis may also collectively contribute to cognitive decline in MASLD patients, leading to depression, anxiety, reduced concentration and processing speed, reduced memory, and sleepiness [10]. Up to 70% of MASLD patients suffer from depression, memory problems, confusion, and poor concentration, greatly reducing their quality of life [11], their ability to perform daily activities, and their work productivity [12].

Liver biopsy remains the definitive method to confirm the MASLD diagnosis, assess disease activity, determine fibrosis stage, and evaluate architectural integrity [13], but it is an invasive procedure, has associated risks, and is limited by sampling variability and subjective interpretation [14]. In this sense, there is a need for non-invasive methods to avoid the use of hepatic biopsy in MASLD diagnosis. Imaging techniques like ultrasonography (US), computed tomography (CT), and magnetic resonance imaging (MRI) offer a non-invasive means of detecting MASLD. Nonetheless, these methods are both time-consuming and costly [15]. Biomarker panels can serve as non-invasive tools to assess therapy response. However, they must be accurate, readily available, cost-effective, and easy to use [16].

For MASLD management, treatment primarily focuses on dietary adjustments, lifestyle changes, weight loss, and addressing the underlying metabolic syndrome. Lifestyle factors, like dietary patterns, have shifted, leading to an increased prevalence of obesity and IR, while lifestyles have become increasingly sedentary [4]. Moreover, unhealthy dietary patterns and lifestyle behaviors exacerbate the inflammatory environment and accelerate cognitive decline [10]. It is important to focus on these interventions because they have shown promising outcomes in managing the condition [17]. Weight loss is deemed a clinically significant goal with a positive impact on MASLD and its risk factors, such as glucose or lipid levels [1]. Additionally, adopting a Mediterranean dietary pattern may offer benefits beyond improving metabolic syndrome, diabetes, and other ailments, potentially aiding in the reversal of fatty liver disease [18].

In this context, there is a need for non-invasive methods that consider a comprehensive set of factors to determine the presence of MASLD after following a dietary intervention. In this regard, the aim of this study was to explain changes in hepatic variables through changes in circulating miRNA levels, inflammatory and biochemical markers, body composition, and psychological status in subjects with MASLD after 6, 12, and 24 months of dietary intervention as well as to determine if different panels of combined biomarkers were able to predict the presence or absence of MALSD after a dietary intervention at each time point.

## 2. Materials and Methods

### 2.1. Study Participants

The current analysis included data from a randomized controlled trial registered as the FLiO (Fatty Liver in obesity) study (www.clinicaltrials.gov, NCT03183193), where 55 subjects presented MASLD diagnosed by ultrasonography and were obese or overweight (BMI between ≥27.5 and <40 kg/m^2^). Participants were randomly assigned to the American Heart Association (AHA) or to the FLiO diet, during a 2-year intervention, with the aim of reaching a weight loss of at least 3% to 5% to improve hepatic steatosis as the AASLD (American Association for the study of Liver Disease) recommends [19], applying an energy restriction of 30% of the total energy requirements. The first strategy (AHA) has a macronutrient distribution of 55% from carbohydrates, 30% from lipids, and 15% from proteins, while the FLiO diet has a macronutrient distribution of 40–45% from carbohydrates, 30–35% from lipids, and 25% from proteins and a high adherence to the Mediterranean Diet. Since no disparities were detected among diets regarding biochemical and anthropometric measures and hepatic variables during the intervention, as demonstrated by Marin-Alejandre et al. [20], the data were consolidated to enhance statistical power. 

The exclusion criteria of the study included subjects with a history of high alcohol consumption (>21 units per week for men and >14 units for women per week), the presence of known liver diseases different from MASLD, a significant weight loss (>3 kg in the last 3 months), endocrine disorders, pharmacological treatments, which could cause hepatic steatosis or alteration in liver tests (immunosuppressants or corticosteroids, among other), active autoimmune diseases, severe psychiatric disorders, the use of weight modifiers, a lack of independence or inability to adhere to the prescribed diet, and difficulties in attending scheduled appointments [20].

This trial was approved by the Research Ethics Committee of the University of Navarra (ref. 54/2015). All procedures were conducted in compliance with the principles outlined in the Declaration of Helsinki and adhered to the guidelines established by CONSORT 2010. All the subjects gave written informed consent prior to their enrollment.

### 2.2. Body Composition, Anthropometric, and Biochemical Determination

At the Metabolic Unit of the University of Navarra, the biochemical determination and body composition were carried out, under fasting conditions, following a described standardized procedure [21]. Body mass index (BMI) was determined by dividing body weight (kg) by the square of the height (m). Body composition, including fat mass and visceral adipose tissue (VAT), was determined by dual-energy X-ray absorptiometry (Lunar iDXA, encore 14.5, Madison, WI, USA).

Blood samples were obtained following 12 h of overnight fasting and then processed and stored at −80 °C until subsequent analyses [21]. Specific commercial kits (Glucose HK CP Ref: A11A01667; Triglycerides CP Ref: A11A01640; HDL Direct CP Ref: A11A01636, ALT CP Ref: A11A01627; AST CP Ref: A11A01629; GGT CP Ref: A11A01630; Cholesterol CP Ref: A11A01634) were used to determine serum biochemical parameters (HORIBA ABX SAS, Montpelier, France) such as blood glucose, triglyceride (TG), high-density lipoprotein cholesterol (HDL-c), alanine aminotransferase (ALT), aspartate aminotransferase (AST), gamma-glutamyl transferase (GGT), and total cholesterol (CT) on an autoanalyzer Pentra C-200, following the instructions of the manufacturer (cobas 8000, Roche Diagnostics, Basel, Switzerland). The Friedewald formula was used to calculate the low-density lipoprotein cholesterol [22]: LDL-c = total cholesterol − HDL-c − TAG/5. To assess insulin, adiponectin, chemerin, retinol-binding protein (RBP4), and leptin concentrations, specific ELISA kits (Human insulin Elisa Mercordia Ref: 10-1113-01; Human Adiponectin Elisa Ref: RD191023100; Human Chemerin Elisa Kit Ref: RD191136200R Biovendor; retinol-binding protein RBP4 ELISA Inmundiagnostik Ref: K6110; Human Leptin Mercodia Ref: 10-119901) were used on a Triturus autoanalyzer (Grifols, Barcelona, Spain) to determine hormones and proteins. The homeostatic model assessment for insulin resistance was calculated using the following formula [21]: HOMA-IR = (glucose (mmol/L) × insulin (μU/mL))/22.5. Leukocyte cell-derived chemotaxin-2 (LECT2) was quantified using the Triturus autoanalyzer, employing specific kits (Human LECT2 Elisa Ref: RD19137200R) designed for this chemotaxin (Biovendor LLC, Asheville, NC, USA). M30 and M65 levels were quantified employing commercial ELISA (M30 Apoptosense Elisa Ref: 10011; M65 Elisa Ref:10020) kits (PEVIVA, Bromma, Sweden) in accordance with the instructions of the manufacturer. Ferritin levels were evaluated using a Chemiluminescent Microparticle Immunoassay (CMIA) technology (Abbott Architect Ferritin Assay) by an external certified laboratory (Eurofins Megalab S.A, Madrid, Spain).

### 2.3. Lifestyle Assessment: Dietary Intake and Physical and Psychological State

Dietary intake was evaluated with a validated semiquantitative 137-item food frequency questionnaire (FFQ) by professional dietitians [23]. For each item in the questionnaire, a typical portion size was provided. Daily food consumption for an item was estimated by multiplying the portion size by the frequency of consumption and dividing, as described [23]. Spanish food composition tables were used to evaluate the nutrient composition of the specific serving size for each food [24]. Adherence to the Mediterranean Diet (MedDiet) was evaluated using a 17-point screening questionnaire. Scores ranged from 0 to 17, with a higher score indicating greater adherence to the MedDiet.

Physical activity levels were evaluated using a validated Spanish version of the Minnesota Leisure-Time Physical Activity Questionnaire [25,26]. The estimation of energy expenditure during the practice of exercise was calculated assuming that 1 MET (Metabolic Equivalent for Task) corresponds to a rate of 3.5 mL/kg/min. Additionally, participants were provided with a step-based physical activity recommendation of 10,000 steps per day [27].

The presence of depressive symptoms was evaluated using the Spanish version of the Beck Depression Inventory-II (BDI-II) [28]. To assess the severity of depressive symptoms is the aim of this questionnaire, which consists of 21 items, in which four response options are presented on a scale from 0 to 3. The total score, ranging from 0 to 63, is derived by summing up the score of all items, with higher scores indicating more pronounced depressive symptomatology.

### 2.4. Imaging Techniques

The hepatic status evaluation was carried out at the University of Navarra Clinic under fasting conditions by qualified professionals. Hepatic steatosis was determined by ultrasonography (Siemens ACUSON S2000 and S3000, Erlangen, Germany) as described elsewhere [29] and clinically classified according to a four-point scale: <5% (grade 0), 5–33% (grade 1), 33–66% (grade 2), and >66% (grade 3) [30]. Hepatic fat content and volume were assessed by MRI (MRI) using the Siemens Aera 1.5 T system, employing the DIXON technique as recommended by the manufacturer [31]. The imaging protocol comprised a DIXON screening sequence for qualitative evaluation of hepatic steatosis, alongside quantitative techniques like multi-echo T2-corrected single breath-hold spectroscopy (HISTO) and multi-echo 3D gradient echo (VIBE) imaging with Dixon reconstruction and T2* correction for semi-quantitative assessments of fat deposition [32,33]. ARFI elastography was employed to measure liver stiffness, with the mean value calculated from 10 valid ARFI measurements for each participant [31].

### 2.5. RNA Isolation, Reverse Transcription, and Real-Time PCR (RT-PCR)

Serum was obtained from whole blood by centrifugation at 1100× *g* at 4 °C for 15 min using the Model 5415R centrifuge (Eppendorf AG, Hamburg, Germany). The samples were subsequently frozen at −80 °C until RNA reverse transcription. Following RNA extraction, total RNA from the serum sample was isolated using the RNeasy Serum/Plasma Advanced Kit (Qiagen, Hilden, Germany), following the manufacturer’s protocol (Cat. No. 217204). The isolation procedure involved the use of absolute ethanol and isopropanol free of nucleases. cDNA synthesis was performed using 4 μL of miRNA sample and the miRCURY LNA RT Kit (Cat. No. 339340) (Qiagen, Hilden, Germany). Subsequently, the protocol for miRNA expression analysis from serum was carried out. Quantitative PCR (qPCR) was conducted using the CFX384 Touch Real-Time PCR system (Bio-Rad, Hercules, CA, USA) with the miRCURY SYBR^®^ Green PCR Kit (Cat. No. 339345) and the following miRCURY LNA miRNA PCR Assays (Cat. No. 339306): Ref. 339306, hsa-miR122-5p, hsa-miR-126-5p, hsa-miR-151a-3p, hsa-miR-192-5p, hsa-miR-15b-3p, hsa-miR-29b-3p, hsa-miR-21-5p, hsa-miR-222-3p (Qiagen, Hilden, Germany). Only assays with a cycle threshold (Cq) value of less than 35 were considered for data analysis [34]. The relative quantities (RQs) of miRNAs were calculated using the formula 2^−ΔCt^, where ΔCt represents the difference between the cycle threshold (Ct) values of the miRNA and the exogenous reference gene UniSp6. The normalization factor (NF) was determined by computing the geometric mean of RQs for all expressed miRNAs in each sample. The normalized relative quantities (NRQ) were obtained by dividing RQs by the sample-specific NF, as described previously [35]. The results were expressed as fold changes (FC) for each miRNA relative to the exogenous reference gene UniSp6, the spike-in used to assess the quality of the cDNA synthesis and qPCR process.

### 2.6. In Silico Evaluation

We conducted an *in silico* analysis to investigate the relationship between miRNAs, validated target genes, and MASLD. Based on scientific literature, we selected those miRNAs that previously had been related to MASLD and its comorbidities [36,37,38]. After identifying our candidate miRNAs, potential target genes of miR122-5p, miR21-5p, miR126-5p, miR151a-3p, miR29b-3p, miR15b-3p, miR222-3p, miR126-5p were obtained using the miRWalk database (http://mirwalk.umm.uni-heidelberg.de/; accessed on 12 May 2024), which uses a self-developed algorithm to predict target genes and makes comparisons with other publicly available databases (TargetScan, miRDB, miRTarBase). Additionally, target genes associated with MASLD pathways were also obtained. For the search, filtering was conducted with a score of 1, and those candidate genes that appeared in at least 2 of three databases were selected. In the case of miR126-5p and miR15b-3p, all genes were included due to the limited number of candidate genes. Duplicates were also discarded (miRNA potentially binding in two sites within the same target gene). Furthermore, to identify metabolic pathways linked to target genes, we utilized GeneCodis (https://genecodis.genyo.es/; accessed on 12 May 2024), focusing on Homo sapiens as the primary organism.

### 2.7. Statistical Analyses

All statistical analyses were performed using Stata version 15.0 (StataCorp 2011, College Station, TX, USA). All p-values reported were two-tailed, with differences considered statistically significant at *p* < 0.05. The normal distribution of the variables was assessed using the Shapiro–Wilk test. Data are presented according to the distribution, either as mean ± standard deviation (SD) or median ± interquartile range (IQR). Group differences were evaluated using Student’s *t*-test or the Mann–Whitney U test for continuous variables and the chi-squared test for categorical variables. The associations between variables were examined using the Pearson correlation coefficient or Spearman’s rho (*p*), as appropriate. The differences between various time points were evaluated using either a paired Student’s *t*-test or a Wilcoxon signed-rank test, depending on their appropriateness for the data.

Multivariable linear regression analyses were performed at each point of the study with changes in hepatic fat content (%) or liver stiffness (m/s) as dependent variables and changes in miRNAs, body composition, inflammatory and biochemical markers, and depressive symptoms as independent variables. All these models are adjusted by sex, age, changes in physical activity, and group of diet. Subsequently, a statistic command (vselect) was used to choose the best combination of independent variables to explain the changes in hepatic fat content and liver stiffness. The generated panels, which included the combinations of independent variables, give a broader understanding of changes in hepatic fat content and liver stiffness in comparison with the utilization of each independent variable individually.

Finally, multivariable logistic regression analyses were conducted with the presence of MASLD as dependent variable, and as independent variables, we considered the panel combinations constructed previously, incorporating the hepatic variables (hepatic fat and liver stiffness) in the model. Receiver Operating Characteristic Curve Analyses (ROC) and areas under the ROC curve (AUROC) were calculated to assess the predictive ability of this combination of panels for MASLD. A higher AUROC value indicates better model performance, and according to Nahm [39], the AUC must be greater than 0.8 to be considered acceptable. For regression analyses, the presence of MASLD was categorized into two groups: subjects who still had steatosis after following a dietary intervention, categorized as grade 1, 2, or 3 (Bad responders = 1) and, conversely, subjects in whom steatosis resolved and who achieved grade 0 (Good responders = 0). The results were validated using the optimism-corrected value with Tibshirani’s enhanced bootstrap method [40].

## 3. Results

### 3.1. Changes in Characteristics after 6, 12, and 24 Months

Characteristics of 55 subjects with MASLD were analyzed at baseline and after 6, 12, and 24 months of dietary intervention. Additionally, differences between the baseline and each time point of the study were examined (Table 1). Significant decreases were observed at all time points in body composition. After 6- and 12-month interventions, total cholesterol, TG, and LDL-c significantly decreased, while HDL-c increased.

Significant improvements in hepatic status were observed after following the dietary intervention, with a significant decrease in the steatosis degree, hepatic fat, and volume content, as well as in liver transaminases. It is noteworthy that this improvement was maintained throughout the 24-month intervention duration of the study. An overview of changes in hepatic status between subjects who still had MASLD (bad responders) after 6, 12, and 24 months of intervention and those who no longer had it (good responders) is shown in Table S1. We emphasize that, in the good responder group, liver stiffness significantly decreases after 6 months. Additionally, steatosis degree significantly decreased in the same group after 6, 12, and 24 months of intervention.

Depression status was assessed using the Beck Depression Inventory questionnaire, revealing a significant decrease in depressive symptoms after 6, 12, and 24 months of dietary intervention. In addition, interactions between changes in depression symptoms and changes in different biomarkers such as leptin, adiponectin, HDL, and miR192-5p were observed (Table S2). Additionally, subjects improved their MedDiet adherence and physical activity after 6, 12, and 24 months of intervention, with a significant decrease in total energy as well as an increase in fiber and protein consumption. Furthermore, the good responder group increased their MedDiet adherence compared to the bad responder group (Table S3).

The adiponectin levels significantly increased after 6, 12, and 24 months of intervention. Conversely, ferritin, RBP4, and leptin levels decreased significantly at each time point of the study, with ferritin values being predominantly reduced after 12 months. Other markers such as chemerin and LECT2 were significantly decreased after 6 and 12 months of intervention. We found that inflammatory variables such as M30 increased at 12 months and M65 decreased at 6 months. Circulating miRNA levels were evaluated after intervention, and we found that miR151a-3p was significantly decreased after 6 months of intervention (Table S4).

### 3.2. Evaluation of the Association of the Changes in Liver Stiffness and Hepatic Fat Content with Changes in miRNAs, Inflammatory, and Biochemical Markers and Depressive Symptoms

As observed in Table 1, most variables decreased after dietary intervention. In this regard, we aimed to investigate whether these changes are influenced by or associated with changes in hepatic fat content and liver stiffness. All linear regression analyses were adjusted by sex, age, physical activity, and diet group (Table 2).

It is noteworthy that after 6 months of intervention, the reduction in biochemical variables such as glucose, insulin, HOMA-IR, circulating miR15b-3p levels, and ferritin were positively associated with the reduction in hepatic fat content. Conversely, the reduction in BMI, weight, and fat mass, circulating levels of miR29b-3p, and the inflammatory variable chemerin, were also positively associated with changes in liver stiffness.

After 12 months of intervention, the decreases in BMI, weight, fat mass, visceral fat, and inflammatory markers such as ferritin and M65 were positively correlated with reductions in hepatic fat content. However, BMI and weight decreases were additionally correlated with changes in liver stiffness, alongside the decrease in chemerin levels and depressive symptoms.

Finally, when analyzing the association between changes in hepatic fat and liver stiffness after 24 months of intervention, we observed that the reduction in visceral fat, weight, and BMI, as well as levels of miR15b-3p and TG, were significantly associated with the reduction in hepatic fat content. However, only the decrease in depressive symptoms and weight at 24 months was positively associated with changes in liver stiffness.

### 3.3. Determination of the Best Variables to Explain Hepatic Fat Content and Liver Stiffness

Once we have identified markers associated with our hepatic variables, we proceeded to create models that allow us to know how they collectively affect hepatic fat content and liver stiffness. We used a STATA command (vselect) that compares and selects those markers that provide us with the best information to explain our hepatic variables of interest (Table 3).

In this sense, we can observe changes in glucose, ferritin, M65, and miR29b-3p and explain the changes in hepatic fat content after 6 months of intervention. Subsequently, after 12 months, changes in ferritin and M65, accompanied by alterations in TG and fat mass account for hepatic fat. Nonetheless, following the 2-year intervention period, the alterations in depressive symptoms, TC, TG, and miR15b-3p emerge as pivotal factors in elucidating our hepatic variable.

Furthermore, it was observed that the model comprising alterations in HOMA, inflammatory variables such as RBP4 and chemerin, along with changes in BMI, significantly elucidated liver stiffness after 6 months of intervention. Following 12 months, a subsequent model was established with changes in chemerin and BMI variables, along with alterations in miR122-5p levels and depressive symptoms. Upon completion of the intervention, a final model was derived explicating changes in liver stiffness, featuring modifications in depressive symptoms, TG, HDL-c, and BMI.

### 3.4. Evaluation of the Presence or Absence of MASLD after a 2-Year Dietary Intervention

To determine the presence or absence of MASLD, logistic regression analyses were conducted using the panel combinations previously established for hepatic fat content and liver stiffness. Additionally, these hepatic variables were incorporated into the current models, as they are integral components of the disease and aid in its accuracy diagnosis (Table 4). After 6 months of intervention, we obtained only one model capable of making predictions if the subjects had MASLD or not with an AUC of 0.76. This model consisted of changes in liver stiffness, HOMA, ferritin, M65, and miR29b-3p after 6 months of intervention.

On the other hand, we were able to predict the presence of MASLD after 12 months of dietary intervention through two models. The first one consisted of the combination of changes in hepatic fat content, ferritin, M65, TG, and fat mass, with an AUC of 0.80. However, the model that best helps us predict the presence of the disease, considering a wider range of markers, is the one formed by changes in liver stiffness, chemerin, depressive symptoms, miR122-5p, and BMI, with an AUC of 0.86.

Finally, after 24 months of intervention, we obtained the best models to predict the disease. One of them, formed by changes in hepatic fat content, TC, TG, miR15b-3Pp, and depressive symptoms, yielded an AUC of 0.89. Additionally, the last model comprised changes in liver stiffness, depressive symptoms, HDL-c, TG, and BMI and provided an AUC of 0.90 to determine if the subjects still suffer from MASLD after a 2-year dietary intervention follow-up.

### 3.5. In Silico Evaluation

The *in silico* analysis was performed to further explore the potential role of miRNA in MASLD. We found that several genes are regulated by our specific microRNAs (Table S5). Some of these genes are involved in numerous signaling pathways such as PI3K-AKT, AMPK, NF-kappa B, or TNFα, insulin, or FOXO, among others (Figure S1). Table S6 shows the genes that miRWalk considers to be involved in MASLD. Table S7 shows the metabolic pathways involved in MASLD according to GeneCodis database. We have highlighted, in Figure S1, those signaling pathways that are also present in Table S7.

## 4. Discussion

The results of the current research showed that the dietary intervention proved to be effective in improving multiple aspects related to hepatic and metabolic health in subjects with MASLD after a follow-up of 6, 12, and 24 months. It is known that the management of MASLD/MASH patients revolves around lifestyle adjustments, dietary modifications, and achieving weight loss [41]. Our results revealed significant improvements in body composition, lipid markers, and hepatic variables as well as increased adherence to the MedDiet. These findings are consistent with the literature, which indicates weight loss and a high adherence to the Mediterranean dietary pattern for managing MASLD [20,42], as it improves hepatic steatosis [43]. A balanced MedDiet combined with caloric restriction can effectively reduce liver stiffness and hepatic fat content as well as improve lipid profiles and anthropometric variables [44,45,46]. Our results indicate lower hepatic fat content after the intervention, and lower liver stiffness in those individuals who no longer have MASLD after intervention, as well as an improvement in lipid profile and HOMA index. Adopting a healthy lifestyle that includes a balanced macronutrient content following a MedDiet pattern and regular exercise might enhance the quality of life and other pathologies related to MASLD, such as breast cancer or sarcopenia [47,48].

Inflammation plays a key role in MASLD, underscoring the importance of pursuing strategies aimed at reducing it [49,50]. Lifestyle interventions, such as hypocaloric diets based on the Mediterranean pattern, are highly effective and result in a reduction in proinflammatory markers [51], such as leptin and RBP4, as our results showed. Other inflammatory markers such as chemerin, ferritin, M65, and LECT2 decreased, while adiponectin increased after the dietary intervention in our study, suggesting a potential role of dietary modifications in modulating systemic inflammation. Chemerin and RBP4 are associated with obesity and metabolic syndrome markers such as BMI, body fat, leptin, lipid profile, blood glucose measurements, and fatty liver [52,53,54]. Similarly, LECT2 mediates obesity-related metabolic disturbances, and its downregulation ameliorates hepatic steatosis [55]. On the other hand, ferritin and M65 have been described as biomarkers for liver cell death. Previous studies showed that M65 could predict MASLD and its severity [56,57]. Furthermore, adiponectin levels have been reduced in MASLD subjects and indirectly predict steatosis, indicating that it could be a key biomarker for the disease [58]. Adiponectin levels also decrease in subjects with obesity [59], and an increase after dietary intervention is a good indicator of health and hepatic improvements.

The occurrence of high levels of pro-inflammatory cytokines, along with elevated levels of cortisol and epinephrine, has been suggested as a potential mechanism linking MASLD with depression. Moreover, MASLD exhibits a close relationship with obesity and T2DM, both of which have been strongly linked to depressive symptoms [60]. Depression has been associated with an increased risk of developing MASLD [61]. A high prevalence of mental health issues such as depression among adults with MASLD has been reported [62]. The subjects of this study decreased their depressive symptoms after 6, 12, and 24 months of dietary intervention, aligned with the improvement in glucose levels and the reduction in body composition variables. Several studies showed a reduction in depressive symptoms after following a balanced dietary intervention [63], sustained physical activity, and adherence to the MedDiet [64]. In addition, changes in depressive symptoms were related to an improvement in HDL-c and adiponectin, which are related to MASLD. Several studies evidenced that the severity of liver diseases was related to psychiatric disorders [10,65]. This fact confirmed our results, showing that depressive symptoms affect and predict the presence of MASLD along with HDL-c, TG, BMI, and liver stiffness in the same combination panel after 24 months of dietary intervention. Similarly, depressive symptoms along with hepatic fat content, miR15b-3p, TC, and TG predict MASLD after 24 months. Inflammation plays a significant role in depression, mainly by triggering microglia and astrocytes in the central nervous system (CNS) and infiltrating peripheral bone marrow cells (BMCs), which release cytokines and chemokines, thus fueling the inflammatory process. Moreover, there is evidence of an abnormal expression of inflammation-related miRNAs in individuals with depression [66]. Due to their ability to regulate various gene networks linked to neuroplasticity and the stress response, along with their movement between the bloodstream and the brain, miRNAs emerge as promising targets for diagnostic and therapeutic strategies in depressive disorders [67].

In addition, among the factors that have been reported to contribute to the development and progression of MASLD, we found epigenetic factors, such as miRNAs. In recent years, it has been reported that miRNA expression profiles are highly associated with various pathological conditions, such as liver, heart, kidney, and autoimmune diseases and some types of cancer [68]. The dysregulation of miRNA expression, caused by internal or external factors, can contribute to the development of metabolic disorders [69]. Alteration in the expression of miRNAs has been linked to MASLD and has been correlated with hepatic steatosis and MASH [70]. While the precise molecular mechanisms and biological pathways driving disease progression remain incompletely understood, genetic variation may explain a portion of the complexity and individual variations observed in the disease phenotype [71]. Several studies have investigated the relationship between pathogenesis and miRNAs in MASH/MASLD [72]. MiRNAs regulate various signaling pathways [73] such as PI3K-AKT, mTOR, FOXO, AMPK, NF-kappa B, or TNFα, among others, which are linked to MASLD development [74,75,76]. These pathways intersect with factors like lipid accumulation, IR, oxidative stress, HSC activation, and inflammatory responses, thereby contributing to MASLD pathogenesis [77]. This study investigated the longitudinal changes in circulating miRNA expression levels as potential indicators of hepatic disease progression. Our findings revealed that circulating miRNA expression was generally maintained over the course of the intervention, except for miR-151a-3p, which exhibited a significant decrease after 6 months of intervention. Moreover, through predictive modeling, miR122-5p and miR15b-3p emerged as potential biomarkers for MASLD presence. These results suggest that alterations in these miRNAs may influence hepatic status in conjunction with other influential variables and have the potential to serve as determinants of disease progression. MiR122-5p was associated with the severity of MASLD and was significantly higher in MASLD subjects compared to healthy subjects [78]. In addition, mir122-5p promotes MASLD progression by modulating Sirt signaling [79]. An elevated miR15b-3p level was found to inhibit cell proliferation and glucose consumption, while also promoting intracellular TG accumulation [80]. Furthermore, a significant increase in circulating miR15b-3p expression was observed in MASLD models [81]. This study sheds light on the utility of circulating miRNAs as non-invasive biomarkers for assessing hepatic health and disease progression.

Finally, we aimed to create diverse panels to determine which combined variables are most suitable for predicting the presence of MASLD, depending on the duration of the dietary intervention. These combinations encompass factors related to MASLD, in order to achieve greater effectiveness, taking into account the influence of each of them. The most effective panels were achieved after a 24-month nutritional intervention. These panels were composed of the combination of changes in circulating miRNAs, general biochemical markers such as TG, TC, HDL-c, and BMI, and psychological factors such as depressive symptoms. Other panels such as HSI, the MASLD liver fat score, and the SteatoTest have been shown to have limitations in accurately predicting hepatic steatosis. They exhibit moderate to low diagnostic power, reduced accuracy in specific populations, reliance on less commonly used tests, and limited ability to discriminate between different levels of steatosis [82,83,84]. Likewise, different miRNA combinations, which included miR-122, miR-1290, miR-27b, and miR-192, in a previous study, showed a strong diagnostic accuracy for MASLD with higher sensitivity and specificity compared to ALT and FIB-4 [85]. A blood-based diagnostic test, named NIS4, was developed and validated to identify or rule out individuals at risk of MASH. The NIS4 algorithm derives from four unique biomarkers associated with MASH (miR-34a-5p, alpha-2 macroglobulin, YKL-40, and glycated hemoglobin) [86]. A circulatory endothelin 1-regulating RNAs panel composed of EDN1/TNF/MAPK3/EP300/hsa-miR-6888-5p/lncRNA RABGAP1L-DT-206 showed positive regulation in patients with MASLD/MASH compared to controls, thus potentially serving as a tool for the early diagnosis and stratification of hepatic fibrosis in MASLD/MASH patients [87].

The results of this study are very promising as they suggest that long-term nutritional interventions aimed at weight loss may have a significant impact on a range of markers related to MASLD. Low-calorie diets (LCD) may reduce body weight and liver fat content in both MASLD and obese subjects, improving liver enzyme levels. Furthermore, Mediterranean-style LCDs, especially green-Mediterranean variants, may independently lower intrahepatic lipid content (IHL) and ALT levels compared to other LCDs [88]. Further insights from dietary intervention trials involving MASLD subjects are crucial to better understanding the dynamics of the contributing factors over time and their potential to differentiate the disease, thus serving as a personalized precision nutrition tool. Additionally, childhood presents a crucial opportunity to instill healthy behaviors, leading to better nutritional and metabolic health during the early stages of development and safeguarding against long-term chronic illnesses [89]. The combination of changes in circulating miRNA, general biochemical markers, and psychological factors offers a comprehensive insight into the effects of the intervention, which may be crucial to understanding and addressing the disease. These findings support the importance of considering multiple factors in the design of intervention strategies for complex metabolic diseases such as MASLD. 

There is an absence of comprehensive studies integrating this extensive array of variables into a unified analytical framework. This highlights the pressing need for systematic investigations that evaluate the complex interplay among these multifaceted factors. Furthermore, there is potential for the development of composite panels that combine optimal variables from each category. Such panels would facilitate a more nuanced understanding of MASLD progression and the effectiveness of intervention strategies across varying timeframes.

This study boasts several notable strengths. Firstly, its considerable duration is a key asset. The extended trial period allows for data collection at multiple time points, facilitating comparisons throughout the study. This longitudinal approach is invaluable in comprehending the dietary effects on MASLD. Additionally, the study participants are meticulously characterized, not only in terms of hepatic parameters but also concerning body composition, biochemical markers, psychological state, and circulating miRNA expression profiles. This comprehensive characterization is attributed to the expertise of the multidisciplinary team, composed of nutritionists, radiologists, internal medicine physicians, technicians, and researchers in the field of precision nutrition. To ensure the homogeneity of the sample, all enrolled subjects came from the same geographical area with similar nutritional habits.

This study has some limitations that need to be acknowledged. It is important to note that the patients in this study came from a specific population and specifically targeted overweight or obese subjects, so the findings may not fully generalize to more diverse populations and may not universally apply to all individuals categorized as obese. Therefore, further population-based studies or multicenter trials are necessary to validate the results in more diverse populations. Secondly, the assessment of liver condition was carried out using non-invasive methods including ultrasonography and magnetic resonance imaging. However, these methods may not offer the comprehensive analysis obtained from a liver biopsy. Thirdly, another limitation is the sample size. More large-sample studies are required to support the above findings in the future. Fourthly, in our study, MASLD subjects consisted of obese individuals with MASLD. It would have been more interesting to have obese controls without MASLD to establish differences between groups. A fifth limitation was that many circulating miRNAs are expressed at low levels, and a threshold of 35 cycles could generate biased results. The use of a high-sensitive platform for precise miRNA quantification (e.g., droplet digital PCR) could be interesting.

## 5. Conclusions

Current findings highlight the potential usefulness of combining circulating miRNAs, inflammatory markers, body composition, general biochemistry, and hepatic variables as predictive tools for MASLD, evolving after following 6, 12, and 24 months of dietary intervention. Moreover, the study underscores the effectiveness of dietary interventions in ameliorating MASLD, emphasizing the importance of precision nutrition and sustained adherence to healthy lifestyle patterns for the management of MASLD.

## Figures and Tables

**Table 1:** Changes in body composition, anthropometric measurements, biochemical determinations, hepatic status, and lifestyle assessment in MASLD subjects after 6, 12, and 24 months of nutritional intervention.

MASLD Group (*n* = 55)				
	Baseline	∆ 6 Months	∆ 12 Months	∆ 24 Months
**Body composition, anthropometric, and biochemical determinations**				
∆ Weight (kg)	94.69 (14.51)	−10.60 (6.05) ***	−8.92 (6.74) ***	−6.11 (6.49) ***
∆ BMI (kg/m^2^)	32.3 (30.2; 35.8)	−3.4 (−5.0; −2.2) ***	−2.8 (−4.8; −1.2) ***	−2.1 (−2.9; −0.6) ***
∆ VAT (kg)	2.2 (1.6; 2.7)	−0.8 (−1.2; −0.3) ***	−0.6 (−1.0; −0.2) ***	−0.3 (−0.7; −0.07) ***
∆ Fat mass (kg)	35.8 (32.24; 41.5)	−7.0 (−11.5; −4.1) ***	−6.0 (−10.9; −2.5) ***	−3.5 (−6.0; −0.5) ***
∆ Glucose (mg/dL)	102.0 (92.0; 109.0)	−8.0 (−18.0; −2.0) ***	−12.0 (−18.0; −2) ***	−8.0 (−17.0; −3.0) ***
∆ Insulin (mU/L)	16.3 (12; 20.9)	−7.6 (−12.6; −1.9) ***	−5.4 (−8.6; −0.2) ***	−5.6 (−9.3; −1.5) ***
∆ HOMA-IR	4.2 (2.9; 5.7)	−2.0 (−3.3; −0.8) ***	−1.5 (−2.5; −0.2) ***	−1.7 (−2.5; −0.8) ***
∆ Total cholesterol (mg/dL)	188.7 (37.1)	−14.0 (−33; 2) **	−12.1 (27.2) **	−0.9 (37.4)
∆ HDL-c (mg/dL)	52.1 (13.9)	0 (−3; 6) *	3.5 (8.1) **	1.7 (12.8)
∆ LDL-c (mg/dL)	110.0 (33.1)	−8.20 (−23.2;7.2) *	−10.0 (25.3) **	0.05 (30.9)
∆ Triglycerides (mg/dL)	123.0 (86.0; 150.0)	−35.7 (64.5) ***	−17.0 (−59.0; 4.0) ***	−14.0 (−39.0; 16.0)
**Hepatic status**				
∆ Steatosis degree	1 (1; 2)	−1 (−1; 0) **	−1 (−1; 0) **	−1 (−1; 0) **
∆ Hepatic fat content (%)	9.2 (5.7; 13.9)	−4.6 (4.34) ***	−3.0 (−6; −1.2) ***	−1.4 (−5.6; 0.1) ***
∆ liver vol. (mL)	1697.0 (1409.0; 2002.0)	−157.6 (192.6) ***	−105.5 (−213.0; 0) ***	−102.5 (−225.0; −3.0) ***
∆ Liver stiffness (m/s)	1.5 (1.2; 2.3)	0.06 (0.9)	0.2 (−0.4; 0.6)	0.09 (−0.5; 0.4)
∆ ALT (IU/L)	30 (21; 43)	−9 (−15; −3) ***	−6 (−13; −2) ***	−4 (−10; 0) ***
∆ AST (IU/L)	24 (19; 28)	−2 (−6; 1) **	−1 (−5; 1) *	0 (−2; 3)
∆ GGT (IU/L)	30 (20; 44)	−10 (−17; −3) ***	−6 (−16; −3) ***	−7 (−15; −2) ***
**Lifestyle assessment: dietary intake, physical activity, and psychological state**				
∆ MedDiet adherence score	6 (4; 7)	6 (4; 9) ***	5 (4; 7) ***	4.36 (3.18) ***
∆ Total energy (kcal/day)	2590 (2154; 2895)	−642 (1067) ***	−653 (996) ***	−522 (−970; 143) **
∆ Carbohydrates (TEV%)	43.77 (7.01)	−1.11 (10.02)	0.056 (10.29)	−2.3 (9.93)
∆ Proteins (TEV%)	16.6 (14.6; 18.7)	2.5 (−0.1; 5.3) ***	1.9 (0.3; 4.9) ***	2.2 (4.7) ***
∆ Lipids (TEV%)	36.1 (6.9)	−2.1 (−8.3; 5.0)	−3.0 (9.5)	0.5 (9.8)
∆ Fiber (g/day)	24.4 (19.6; 30.2)	6.3 (11.6) *	6.2 (14.3) **	2.5 (−1.4; 13.4) **
∆ PA (METs-min/week)	2238 (1665; 4295)	1010 (−120; 2400) ***	487.25 (−608; 2038) *	490 (−826; 2175) *
∆ Beck	6 (2; 9)	−3 (−4; 0) ***	−2.85 (5.66) ***	−2 (−4; 0) ***
**Inflammatory markers**				
∆ Ferritin (ng/mL)	95.5 (48.0; 223.7)	−12.28 (−51.2; 0.9) ***	−49.9 (−135.8; −21.1) ***	−14.6 (−58.5; 14.2) ***
∆ Chemerin (ng/mL)	210.9 (34.4)	−17.8 (40.9) **	−25.4 (41.6) ***	12.0 (55.1)
∆ LECT2 (ng/mL)	43.4 (38.2; 46.8)	−5.3 (9.1) ***	−4.6 (9.6) ***	0.8 (10.6)
∆ RBP4 (mg/L)	33 (28.2; 41.6)	−8.8 (−16.1; −1.7) ***	−10.4 (9.1) ***	−10.5 (9.6) ***
∆ Leptin (ng/mL)	26.8 (15.8; 41.4)	−9.6 (−16.4; −4.9) ***	−9.1 (−18.1; −1.8) ***	−4.4 (−12.4; 2.5) **
∆ Adiponectin (μg/mL)	7.3 (2.2)	1.1 (−0.6; 3.4) ***	1.5 (3.6) ***	2.1 (3.5) ***
∆ M30 (U/L)	72.8 (38.8; 116.3)	−12.1 (60.7)	53.7 (−19.2; 84.3) ***	5.1 (−32.1; 48.9)
∆ M65 (U/L)	(88.1; 187.2)	−41.1 (−87.5; 0.13) ***	−20.4 (−67.7; 20.9)	−10.2 (−59.1; 32.3)

Values are expressed as mean (SD) or median (IQR), according to their distribution. Abbreviations: MASLD, metabolic dysfunction-associated steatotic liver disease; BMI, body mass index; VAT, visceral adipose tissue; HOMA-IR, homeostatic model assessment for insulin resistance; HDL-c, high-density lipoprotein cholesterol; LDL-c, low-density lipoprotein cholesterol; ALT, alanine aminotransferase; AST, aspartate aminotransferase; GGT, gamma-glutamyl transferase; PA, physical activity; MedDiet, Mediterranean Diet; LECT2, leukocyte cell-derived chemotaxin-2; RBP4, retinol-binding protein. miR, microRNA. Comparison between baseline vs 6, 12 and 24 months: * *p* < 0.05, ** *p* < 0.01, *** *p* < 0.001. ∆ Changes between baseline and after 6, 12, and 24 months.

**Table 2:** Multiple linear regression model of changes in hepatic fat content and liver stiffness (dependent variables) with changes in circulating miRNAs, body composition, anthropometric, and biochemical determinations, and inflammatory markers at different points of the study.

	∆ 6 Months				∆ 12 Months				∆ 24 Months			
MASLD (*n* = 55)	∆ Hepatic Fat Content (%)		∆ Liver Stiffness (m/s)		∆ Hepatic Fat Content (%)		∆ Liver Stiffness (m/s)		∆ Hepatic Fat Content (%)		∆ Liver Stiffness (m/s)	
	β	*p*-Value	Β	*p*-Value	β	*p*-Value	β	*p*-Value	β	*p*-Value	β	*p*-Value
∆ miR21-5p (FC)	0.098	0.833	0.170	0.221	0.113	0.730	−0.061	0.395	0.286	0.279	0.022	0.531
∆ miR151a-3p (FC)	0.290	0.483	0.127	0.260	0.135	0.573	−0.031	0.542	0.143	0.432	0.0166	0.489
∆ miR192-5p (FC)	0.626	0.098	0.143	0.222	0.148	0.637	−0.036	0.593	0.192	0.465	−0.009	0.784
∆ mir15b-3p (FC)	0.790	0.015	0.109	0.293	0.159	0.417	−0.027	0.518	0.905	0.014	0.037	0.429
∆ mir29b-3p (FC)	0.416	0.173	0.215	0.017	0.120	0.623	−0.044	0.418	0.671	0.054	0.036	0.411
∆ miR126-5p (FC)	−0.004	0.973	0.016	0.638	−0.064	0.640	−0.014	0.634	0.320	0.164	0.021	0.504
∆ mir222-3p (FC)	0.165	0.545	0.073	0.317	0.263	0.337	0.0030	0.961	0.317	0.126	0.008	0.776
∆ mir122-5p (FC)	0.063	0.828	0.026	0.752	0.422	0.099	−0.073	0.171	0.075	0.753	−0.003	0.913
∆ Weight (kg)	0.058	0.564	0.059	0.015	0.207	0.047	0.042	0.042	0.296	0.011	0.032	0.043
∆ BMI (kg/m^2^)	0.251	0.890	0.163	0.017	0.665	0.025	0.119	0.049	0.861	0.010	0.090	0.050
∆ VAT (kg)	2.034	0.087	0.343	0.258	2.502	0.022	0.175	0.477	2.652	0.030	0.131	0.469
∆ Fat mass (kg)	0.130	0.245	0.063	0.023	0.331	0.006	0.045	0.054	0.204	0.102	0.026	0.138
∆ Depressive symptoms	0.153	0.132	0.038	0.120	0.165	0.134	0.028	0.198	0.202	0.199	0.042	0.046
∆ Glucose (mg/dL)	0.125	0.023	−0.010	0.455	0.036	0.450	0.0008	0.936	0.073	0.154	−0.002	0.684
∆ Insulin (mU/L)	0.228	0.005	−0.018	0.372	0.150	0.122	0.019	0.337	0.177	0.110	−0.004	0.777
∆ HOMA-IR	0.793	0.005	−0.108	0.130	0.554	0.101	0.032	0.622	0.556	0.144	−0.039	0.447
∆ TC (mg/dL)	−0.012	0.503	−0.0004	0.927	0.0001	0.994	0.002	0.619	0.033	0.074	−0.0007	0.784
∆ HDL-c (mg/dL)	0.009	0.901	0.0181	0.324	0.021	0.777	0.007	0.617	0.028	0.616	−0.009	0.206
∆ LDL-c (mg/dL)	−0.007	0.730	−0.0007	0.886	0.020	0.409	−0.0007	0.885	0.024	0.298	−0.001	0.627
∆ TG (mg/dL)	−0.010	0.295	−0.001	0.595	−0.020	0.050	0.002	0.286	0.024	0.014	0.002	0.085
∆ Ferritin (ng/mL)	0.037	0.008	−0.004	0.233	0.021	0.015	0.001	0.344	0.012	0.359	−0.001	0.597
∆ Chemerin (ng/mL)	−0.006	0.683	0.009	0.009	0.012	0.374	0.007	0.007	0.001	0.907	−0.0000	0.979
∆ LECT2 (ng/mL)	0.046	0.483	0.017	0.271	0.052	0.420	0.001	0.886	−0.085	0.206	−0.007	0.433
∆ RBP4 (mg/L)	−0.040	0.470	0.019	0.169	−0.046	0.484	−0.003	0.814	−0.031	0.688	0.0047	0.660
∆ Leptin (ng/mL)	0.021	0.426	0.003	0.614	0.017	0.490	0.004	0.450	0.011	0.714	2.209	0.138
∆ Adiponectin (μg/mL)	0.167	0.235	−0.001	0.973	0.062	0.688	0.0181	0.602	0.190	0.372	0.007	0.806
∆ M30 (U/L)	0.004	0.676	0.005	0.059	0.005	0.418	−0.002	0.110	−0.002	0.811	0.0005	0.736
∆ M65 (U/L)	0.009	0.178	0.003	0.054	0.0136	0.020	−0.0008	0.516	−0.0006	0.943	0.0007	0.545

Abbreviations: MASLD, metabolic dysfunction-associated steatotic liver disease; miR, microRNA; FC, fold change; BMI, body mass index; VAT, visceral adipose tissue; HOMA-IR, homeostatic model assessment for insulin resistance; TG, triglycerides; TC, total cholesterol; HDL-c, high-density lipoprotein cholesterol; LDL-c, low-density lipoprotein cholesterol; MedDiet, Mediterranean Diet; LECT2, leukocyte cell-derived chemotaxin-2; RBP4, retinol-binding protein. ∆ Changes between baseline and after 6, 12, and 24 months. Adjusted by sex, age, PA, and diet group.

**Table 3:** Multiple linear regression model for hepatic fat content and liver stiffness changes as dependent variables at each point of the study.

MASLD (*n* = 55)							
**∆ 6 months**							
∆ Hepatic fat content (%)				∆ Liver stiffness (m/s)			
Model	Adj-R squared	*p*-value ^a^	95% coef. Interval	Model	Adj-R squared	*p*-value ^a^	95% coef. Interval
	0.3997	0.0000			0.3551	0.0001	
	β	*p*-value			β	*p*-value	
∆ Glucose (mg/dL)	0.1499	0.001	0.068; 0.231	∆ HOMA-IR	−0.2061	0.001	−0.322; −0.089
∆ Ferritin (ng/mL)	0.0344	0.005	0.010; 0.057	∆ RBP4 (mg/L)	0.0147	0.106	−0.003; 0.032
∆ M65 (U/L)	0.0124	0.031	0.001; 0.023	∆ Chemerin (ng/mL)	0.0075	0.033	0.0006; 0.014
∆ mirR9b-3p (FC)	0.3883	0.080	−0.048; 0.825	∆ BMI (kg/m^2^)	0.1726	0.010	0.043; 0.301
**∆ 12 months**							
∆ Hepatic fat content (%)				∆ Liver stiffness (m/s)			
Model	Adj-R squared	*p*-value ^a^	95% coef. Interval	Model	Adj-R squared	*p*-value ^a^	95% coef. Interval
	0.3931	0.000			0.1769	0.0093	
	β	*p*-value			β	*p*-value	
∆ Ferritin (ng/mL)	0.0184	0.005	0.005; 0.030	∆ Chemerin (ng/mL)	0.0070	0.013	0.001; 0.012
∆ M65 (U/L)	0.0082	0.085	−0.001; 0.017	∆ Depressive symptoms	0.0185	0.344	−0.020; 0.057
∆ TG (mg/dL)	−0.0178	0.035	−0.034; −0.001	∆ miR122-5p (FC)	−0.0768	0.097	−0.168; 0.014
∆ Fat mass (kg)	0.2701	0.008	0.073; 0.466	∆ BMI (kg/m^2^)	0.0467	0.377	−0.058; 0.152
**∆ 24 months**							
∆ Hepatic fat content (%)				Liver stiffness (m/s)			
Model	Adj-R squared	*p*-value ^a^	95% coef. Interval	Model	Adj-R squared	*p*-value ^a^	95% coef. Interval
	0.4415	0.0001			0.1191	0.0363	
	β	*p*-value			β	*p*-value	
∆ TC (mg/dL)	0.0476	0.007	0.013; 0.081	∆ Depressive symptoms	0.0447	0.024	0.006; 0.083
∆ miR15b-3p (FC)	0.6886	0.004	0.238; 1.138	∆ HDL-c (mg/dL)	−0.0113	0.153	−0.028; −0.0007
∆ TG (mg/dL)	0.0307	0.001	0.013; 0.048	∆ TG (mg/dL)	0.0010	0.511	−0.002; 0.004
∆ Depressive symptoms	0.1705	0.180	−0.082; 0.423	∆ BMI (kg/m^2^)	0.0157	0.751	−0.083; 0.115

Abbreviations: MASLD, metabolic dysfunction-associated steatotic liver disease; m/s, meters per second; miR, microRNA; FC, fold change; BMI, body mass index; HOMA-IR, homeostatic model assessment for insulin resistance; RBP4, retinol-binding protein; TG, triglycerides; CT, total cholesterol; HDL-c, high-density lipoprotein cholesterol. ^a^ *p*-value of the model. ∆ Changes between baseline and after 6, 12, and 24 months.

**Table 4:** Receiver Operating Characteristic Curves for the presence of MASLD after dietary intervention.

MASLD (*n* = 55)					
**∆ 6 months**					
Model 1	*p*-value	AUROC	Model 2	*p*-value	AUROC
∆ Hepatic fat content (%)	0.1733	0.7222 (0.6122 ^†^)	∆ Liver stiffness (m/s)	0.0016	0.8295 (0.7658 ^†^)
∆ Glucose (mg/dL)			∆ HOMA-IR		
∆ Ferritin (ng/mL)			∆ RBP4 (mg/L)		
∆ M65 (U/L)			∆ Chemerin (ng/mL)		
∆ miR29b-3p (FC)			∆ BMI (kg/m^2^)		
**∆ 12 months**					
Model 1	*p*-value	AUROC	Model 2	*p*-value	AUROC
∆ Hepatic fat content (%)	<0.001	0.8550 (0.8029 ^†^)	∆ Liver stiffness (m/s)	<0.001	0.9018 (0.8612 ^†^)
∆ Ferritin (ng/mL)			∆ Chemerin (ng/mL)		
∆ M65 (U/L)			∆ Depressive symptoms		
∆ TG (mg/dL)			∆ miR122-5p (FC)		
∆ Fat mass (kg)			∆ BMI (kg/m^2^)		
**∆ 24 months**					
Model 1	*p*-value	AUROC	Model 2	*p*-value	AUROC
∆ Hepatic fat content (%)	<0.001	0.9495 (0.8996 ^†^)	∆ Liver stiffness (m/s)	<0.001	0.9313 (0.9019 ^†^)
∆ TC (mg/dL)			∆ Depressive symptoms		
∆ miR15b-3p (FC)			∆ HDL-c (mg/dL)		
∆ TG (mg/dL)			∆ TG (mg/dL)		
∆ Depressive symptoms			∆ BMI (kg/m^2^)		

Abbreviations: MASLD, metabolic dysfunction-associated steatotic liver disease; m/s, meters per second; AUROC, area under the Receiver Operating Characteristic Curve; miR, microRNA; m/s, meters per second; FC, fold change; BMI, body mass index; TG, triglycerides; HOMA-IR, homeostatic model assessment for insulin resistance; RBP4, retinol-binding protein; TC, total cholesterol; HDL-c, high-density lipoprotein cholesterol. ^†^ Optimism corrected AUROC value. ∆ Changes between baseline and after 6, 12, and 24 months.

## Data Availability

The data presented in this study are available on request from the corresponding author. The data are not publicly available due to ethical reasons. All the data belong to a private collection named “The FLiO Study”.

## Supplementary Materials

The following supporting information can be downloaded at: https://www.mdpi.com/article/10.3390/nu16111547/s1. Table S1. Characteristics of hepatic status between good and bad responder groups after 6-, 12-, and 24-month dietary intervention. Table S2. Linear regression models for changes in depressive symptoms (dependent variable) and changes in inflammatory markers, miRNAs, body composition, anthropometrics, and biochemical parameters (independent variables) at each point of the study. Table S3. MedDiet adherence score between good and bad responder groups after a 6-, 12-, and 24-month dietary intervention. Table S4. Circulating miRNAs expression level after a 6-, 12-, and 24-month dietary intervention. Table S5. Potential target genes of miRNA candidates using miRWalk database (http://mirwalk.umm.uni-heidelberg.de/, accessed on 12 May 2024), which uses a self-developed algorithm to predict target genes and compares them with other publicly available databases (TargetScan, miRDB, miRTar-Base). Candidate genes that appeared in at least two of three databases were selected. In the case of miR126-5p and miR15b-3p, all genes were included due to the limited number of candidate genes. Table S6. Potential target genes associated with MASLD pathways using miRWalk database (http://mirwalk.umm.uni-heidelberg.de/, accessed on 12 May 2024), which uses a self-developed algorithm to predict target genes and compares them with other publicly available databases (TargetScan, miRDB, miRTar-Base). Table S7. Metabolic pathways associated with MASLD, according to GeneCodis (https://genecodis.genyo.es/, accessed on 12 May 2024) and with *Homo sapiens* chosen as a main organism, when taking into account the target genes associated with the disease obtained from miRWalk ((http://mirwalk.umm.uni-heidelberg.de/, accessed on 12 May 2024). Figure S1. Pathway enrichment of putative candidates of miR151a-3p (A), miR126-5p (B), miR29b-3p (C), miR192-5p (D), miR122-5p (E), miR21-5p (F), miR222-3p (G), and miR15b-3p (H). Red intensity represents number of gene candidates aligned with each pathway description. The top 10 metabolic pathways according to the p-adjusted value were depicted for each miRNA. Those metabolic pathways that are also present in Table S7.
