# Supplementary material for: Multipanel Approach including miRNAs, Inflammatory Markers, and Depressive Symptoms for Metabolic Dysfunction-Associated Steatotic Liver Disease Diagnosis during 2-Year Nutritional Intervention
Source: Nutrients. 2024 May 21;16(11):1547. doi: 10.3390/nu16111547 (PMC11174705; doi:10.3390/nu16111547)
Supplement: Supplementary file 1 [file nutrients-16-01547-s001.zip › nutrients-3002896-supplementary.pdf]

Table S1. Characteristics of hepatic status between good and bad responder groups after 6-, 12- and 24-month dietary intervention.

MASLD (n=55)	Δ 6 months			
	**MASLD (n=55)**	**Good responder group (n=19)**	**Bad responder group (n=31)**	**p-value**
Δ Steatosis degree	-1 (-1; 0) **	-1 (-1; -1)	0 (-1; 0)	<0.001
Δ Hepatic fat content (%)	-4.65 (4.34) ***	-3.57 (3.3)	-5.42 (4.9)	0.1565
Δ Liver vol. (ml)	-157.61 (192.65) ***	-115.94 (158.2)	-191.85 (215.6)	0.2077
Δ Liver stiffness (m/s)	0.059 (0.94)	-0.30 (0.9)	0.29 (0.9)	0.0275
Δ ALT (IU/L)	-9 (-15; -3) ***	-11 (-16; -2)	-7 (-14; -3)	0.5348
Δ AST (IU/L)	-2 (-6; 1) **	-2 (-4; 1)	-2 (-7; 1)	0.7260
Δ GGT (IU/L)	-10 (-17; -3) ***	-11 (-37; -5)	-8 (-14; -1)	0.0854
**MASLD (n=55)**	**Δ 12 months**			
	**MASLD (n=55)**	**Good responder group (n=22)**	**Bad responder group (n=33)**	**p-value**
Δ Steatosis degree	-1 (-1; 0) **	-1 (-1; -1)	0 (-1; 0)	<0.001
Δ Hepatic fat content (%)	-3 (-6; -1.2) ***	-3.25 (-6.1; -1.7)	-2.6 (-6; -0.7)	0.4934
Δ Liver vol. (ml)	-105.5 (-213; 0) ***	-110 (-208; -31)	-101 (-213; 11)	0.7308
Δ Liver stiffness (m/s)	0.16 (-0.46; 0.66)	-0.13 (-1.01; 0.5)	0.24 (-0.11; 0.8)	0.0532
Δ ALT (IU/L)	-6 (-13; -2) ***	-6 (-16; -3)	-6 (-11; 0)	0.6670
Δ AST (IU/L)	-1 (-5; 1) *	-3 (-5; 0)	-1 (-5; 1)	0.3792
Δ GGT (IU/L)	-6 (-16; -3) ***	-8 (-22; -3)	-5 (-10; -3)	0.3307
**MASLD (n=55)**	**Δ 24 months**			
	**MASLD (n=55)**	**Good responder group (n=26)**	**Bad responder group (n=29)**	**p-value**
Δ Steatosis degree	-1 (-1; 0) **	-1 (-1; -1)	0 (-1; 0)	<0.001
Δ Hepatic fat content (%)	-1.4 (-5.6; 0.1) ***	-2.4 (-6.5; 0.5)	-0.55 (-4.15; 1.3)	0.0626
Δ Liver vol. (ml)	-102.5 (-225; -3) ***	-102 (-225; -4)	-129 (-196; -3)	1.000
Δ Liver stiffness (m/s)	0.09 (-0.48; 0.39)	0.07 (-0.7; 0.3)	0.115 (-0.225; 0.6)	0.0548
Δ ALT (IU/L)	-4 (-10; 0) ***	-5.5 (-15; -1)	-3 (-9; 0)	0.3711
Δ AST (IU/L)	0 (-2; 3)	0 (-2; 2)	1 (-2; 3)	0.4464
Δ GGT (IU/L)	-7 (-15; -2) ***	-8 (-21; -4)	-5 (-15; 0)	0.0889

Values are expressed as mean (SD) or median (IQR), according to their distribution. Abbreviations: MASLD, Metabolic dysfunction-associated steatotic liver disease; ALT, Alanine aminotransferase; AST, Aspartate aminotransferase; GGT, Gamma-glutamyl transferase. Comparison between baseline vs 6, 12 and 24 months: *p<0.05, **p<0.01, ***p<0.001. **Δ** Changes between baseline and after 6, 12 and 24 months. Good responders: subjects whose steatosis resolved after following the dietary intervention. Bad responders: subjects who still had steatosis after following the dietary intervention.

Table S2. Linear regression models for changes in depressive symptoms (dependent variable) and changes in inflammatory markers, miRNAs, body composition and anthropometrics and biochemical parameters (independent variables) at each point of the study.

MASLD (n=55)	Depressive symptoms					
	Δ 6 months		Δ 12 months		Δ 24 months	
	β	p-value	β	p-value	β	p-value
Δ Ferritin (ng/ml)	0.0179	0.379	0.0106	0.658	0.0071	0.594
Δ Chemerin (ng/ml)	0.0270	0.195	-0.0318	0.387	0.0048	0.722
Δ LECT2 (ng/ml)	0.0801	0.379	-0.0167	0.920	0.0025	0.968
Δ RBP4 (mg/l)	0.0046	0.954	-0.2018	0.261	-0.0480	0.523
Δ Leptin (ng/mL)	-0.0094	0.803	-0.2519	0.000	0.0452	0.140
Δ Adiponectin (μg/mL)	0.4150	0.034	-0.4810	0.256	0.3024	0.135
Δ M30 (U/L)	-0.0110	0.432	0.0056	0.767	0.0052	0.617
Δ M65 (U/L)	-0.0019	0.839	-0.0073	0.661	0.0067	0.414
Δ miR21-5p (FC)	0.8055	0.214	-0.0496	0.955	-0.3159	0.191
Δ miR151a-3p (FC)	0.2507	0.666	-0.0776	0.908	-0.1158	0.504
Δ miR192-5p (FC)	0.1984	0.715	-0.0456	0.958	-0.4878	0.043
Δ mir15b-3p (FC)	0.7218	0.078	0.1524	0.773	-0.1900	0.584
Δ mir29b-3p (FC)	0.5716	0.218	-0.5074	0.448	-0.1870	0.564
Δ miR126-5p (FC)	0.1054	0.591	-0.3362	0.377	-0.2959	0.167
Δ mir222-3p (FC)	-0.0380	0.921	-0.3560	0.637	-0.3165	0.100
Δ mir122-5p (FC)	0.0280	0.945	-0.3891	0.578	-0.3361	0.125
Δ Glucose (mg/dL)	0.0724	0.360	-0.0907	0.459	-0.0141	0.774
Δ Insulin (mU/L)	0.1494	0.189	-0.2445	0.319	0.0706	0.503
Δ HOMA-IR	0.4407	0.250	-0.6350	0.430	0.0780	0.831
Δ TC (mg/dL)	0.0235	0.380	0.0348	0.565	0.0242	0.181
Δ HDL-c (mg/dL)	0.2327	0.016	-0.2820	0.133	0.0446	0.414
Δ LDL-c (mg/dL)	0.0135	0.652	0.0592	0.358	0.0297	0.180
Δ TG (mg/dL)	-0.0059	0.652	0.0119	0.660	0.0039	0.695
Δ Weight (kg)	0.1203	0.3818	0.2518	0.0636	0.1880	0.1694
Δ BMI (kg/m2)	0.4347	0.285	0.5764	0.149	0.4850	0.135
Δ VAT (kg)	2.6700	0.108	2.4400	0.125	1.963	0.118
Δ Fat mass (Kg)	0.1959	0.217	0.3017	0.052	0.2128	0.091

Abbreviations: MASLD, Metabolic dysfunction-associated steatotic liver disease; LECT2, Leukocyte cell-derived chemotaxin-2; RBP4, Retinol Binding Protein. miR, microRNA; FC, Fold Change; HOMA-IR, Homeostatic model Assessment for Insulin Resistance; TC, Total Cholesterol; HDL-c, High Density Lipoprotein Cholesterol; LDL, Low Density Lipoprotein Cholesterol; TG, Triglycerides; BMI, Body Mass Index; VAT, Visceral Adipose Tissue. Adjusted by sex, aged, physical activity and diet group. Δ Changes between baseline and after 6, 12 and 24 months.

Table S3. MedDiet adherence score between good and bad responder groups after a 6-, 12- and 24-month dietary intervention.

	MASLD (n=55)	Good responder group (n=19)	Bad responder group (n=31)	p-value
	**Δ 6 months**			
Δ MedDiet Adherence score	6 (4; 9) ***	6.89 (3.14)	5.83 (3.36)	0.2754
	**Δ 12 months**			
	5 (4; 7) ***	6.36 (3.06)	4.56 (2.82)	0.0134
	**Δ 24 months**			
	4.36 (3.18) ***	5.30 (3.28)	3.51 (2.88)	0.0359

Abbreviation: MASLD, Metabolic dysfunction-associated steatotic liver disease; MedDiet, Mediterranean Diet. Δ Changes between baseline and after 6, 12 and 24 months of intervention. Good responders: subjects whose steatosis resolved after following the dietary intervention. Bad responders: subjects who still had steatosis after following the dietary intervention.

Table S4. Circulating miRNAs expression level after a 6-, 12- and 24-month dietary intervention.

**MASLD (n=55)**				
**Circulating miRNAs**	**Baseline**	**Δ 6 months**	**Δ 12 months**	**Δ 24 months**
Δ miR21-5p (FC)	1.55 (1.01; 2.34)	-0.45 (-0.84; 0.32)	-0.13 (-1.40; 0.55)	-0.26 (-1.25; 1.92)
Δ miR151a-3p (FC)	1.82 (0.93; 2.74)	-0.29 (-1.38; 0.32) *	-0.39 (-1.45; 0.46)	-0.34 (-1.43; 0.91)
Δ miR192-5p (FC)	1.25 (0.79; 2.15)	-0.19 (-0.74; 0.45)	-0.13 (-0.85; 0.51)	-0.24 (-0.8; 1.31)
Δ mir15b-3p (FC)	1.49 (0.69; 2.39)	-0.17 (-0.85; 0.41)	-0.33 (-1.60; 0.81)	-0.1 (-1.08; 0.74)
Δ mir29b-3p (FC)	1.8 (0.67-2.73)	-0.05 (-1.04; 0.60)	-0.05 (-1.76; 0.63)	-0.49 (-1.42; 1.52)
Δ miR126-5p (FC)	1.86 (0.75; 3.32)	-0.33 (-1.43; 0.42)	-0.26 (-1.82; 0.37)	-.28 (-1.7; 1.85)
Δ mir222-3p (FC)	1.62 (0.51; 2.95)	-0.24 (-1.33; 0.71)	0 (-1.88; 0.61)	0 (-1.51; 1.38)
Δ mir122-5p (FC)	1.57 (0.66; 3.46)	-0.43 (-1.72; 0.57)	-0.31 (-2.06; 0.86)	-0.59 (-2.28; 0.76)

Values are expressed as median (IQR). Abbreviations: MASLD, Metabolic dysfunction-associated steatotic liver disease; miR, microRNA, FC: Fold Change. Comparison between baseline vs 6, 12 and 24 months: *p<0.05. **Δ** Changes between baseline and after 6, 12 and 24 months.

Table S5. Potential target genes of miRNA candidates using miRWalk database (http://mirwalk.umm.uni-heidelberg.de/ accessed on 12 May 2024) which uses a self-developed algorithm to predict target genes and compares to other publicly available databases (TargetScan, miRDB, miRTarBase). Candidate genes that appeared in at least 2 of three databases were selected. In the case of miR126-5p and miR15b-3p all genes were included due to the limited number of candidate genes.

Hsa-miR-122-5p	Hsa-mir-151a-3p	Hsa-mir-21-5p	Hsa-mir-126-5p
DDR2	*FXR1*	*DDAH1*	*HECW2*
SLC52A2	*CYLD*	*SOX5*	*NFIB*
NPEPPS	*NIPAL2*	*RALGPS2*	*TBC1D19*
RIMKLA	*SIX1*	*XKR6*	*OGT*
FOXO3	*PITPNA*	*NEGR1*	*AMMECR1L*
DLAT	*TNPO1*	*ANKRD46*	*FARP1*
RPL17C18orf32	*STXBP4*	*SATB1*	*TEAD1*
HECW2	*MEGF9*	*PPP1R3B*	*LRCH1*
CYP20A1		*DNM1L*	*ELAVL2*
		GID4	*RAB3GAP2*
		NFIA	*SKAP2*
		KLF6	*NRXN1*
		ZNF217	*TBC1D20*
		STRN	*PGM3*
		ATP2B4	
		LMBR1	
		FAM126B	
		ZNF207	
		AGO2	
		EIF4EBP2	

Hsa-mir-29b-3p	Hsa-mir-222-3p	Hsa-mir-15b-3p	Hsa-mir-192-5p
COL4A5	*BBC3*	*KIDINS220*	*DBT*
DNMT3A	*DCAF7*	*GPATCH2*	*ACVR2A*
DYNLT1	*DICER1*	*DNM3*	*LPAR4*
ENPP2	*ESR1*	*CCSER1*	*FRMD4B*
FBRS	*GRB10*	*ST6GALNAC3*	*ZBTB34*
HMGCR	*HIPK2*	*CD47*	*AP3M2*
LAMA2	*PANK3*	*FAM110B*	*NKAIN2*
MAP2K6	*PHACTR4*	*PHF14*	*RB1*
MXD1	*PPP2R2A*	*PTEN*	*B3GALNT1*
NASP	*RNPS1*	*LSM8*	*ANAPC10*
NREP	*STOX2*	*PNMA8A*	*NOD2*
PHACTR2	*TLE3*	*SEPTIN11*	
PPIC	*TMCC1*	*RUNX1T1*	
REL	*TP53BP2*	*ANKFY1*	
TMTC3	*TRPS1*	*DCAF10*	
ZBTB34	*UBN2*	*COBL*	
	ZFYVE16	*CIAO2A*	
	ZNF652	*PHC3*	
		TMEM47	
		FER	

RAB3B
STRN
SLC1A1
STYX
DCTN4
MINK1
ST6GAL2
ARHGAP18

Abbreviations: miR, microRNA; Hsa, *Homo Sapiens.*

Figure S1. Pathway enrichment of putative candidates of miR151a-3p (A), miR126-5p (B), miR29b-3p (C), miR192-5p (D), miR122-5p (E), miR21-5p (F), miR222-3p (G) and miR15b-3p (H). Red intensity represents number of gene candidates aligned with each pathway description. The top 10 metabolic pathways according to p-adjusted value were depicted for each miRNA. Those metabolic pathways that are also present in Table 7S are highlighted in red.

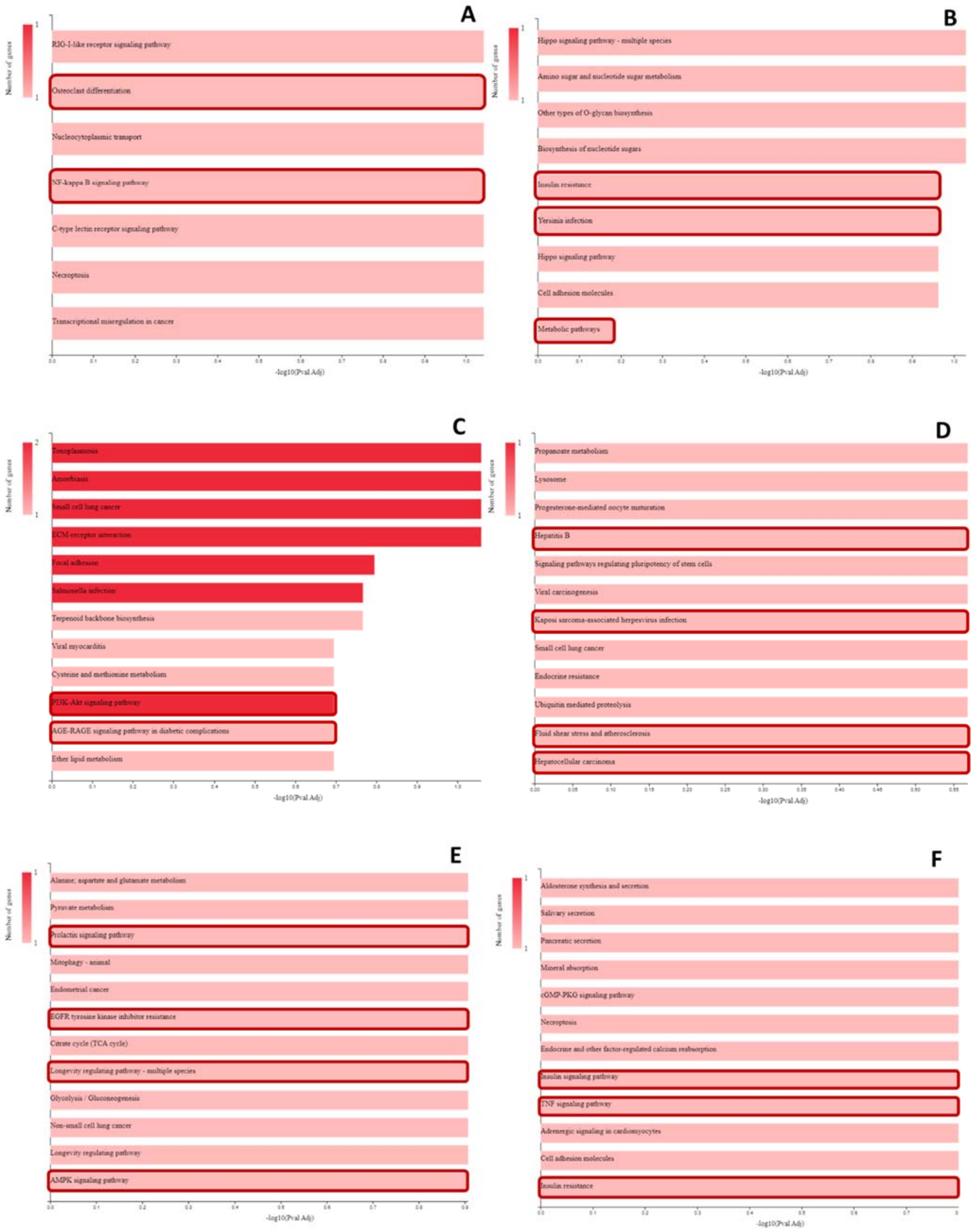

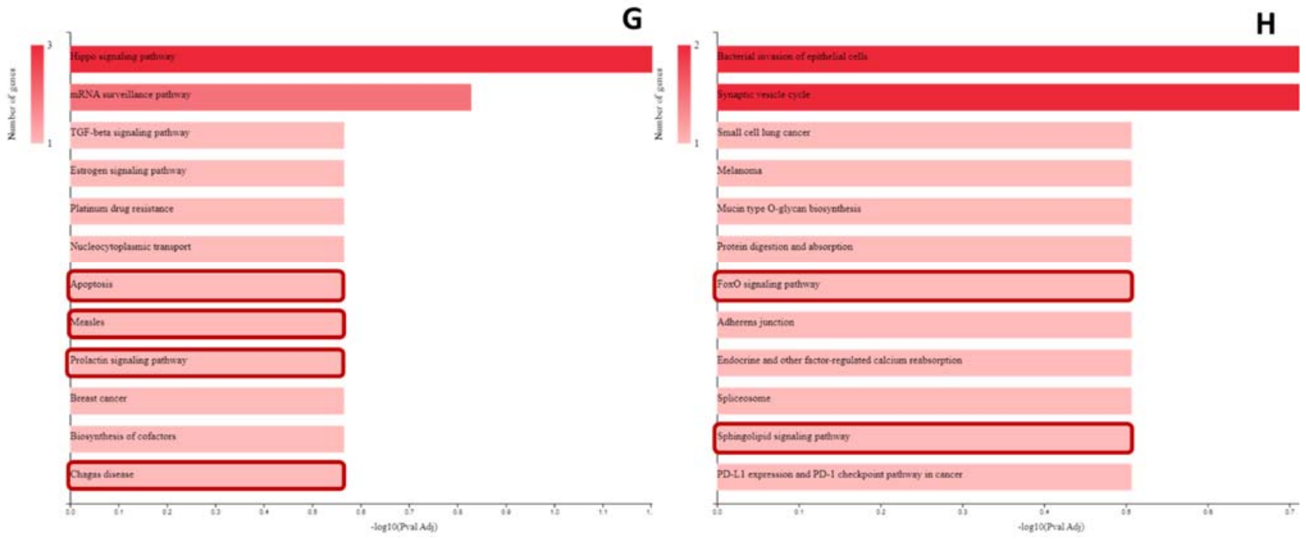
G
Number of genes
3
1
Hippo signaling pathway
mRNA surveillance pathway
TGF-beta signaling pathway
Estrogen signaling pathway
Platinum drug resistance
Nucleocytoplasmic transport
Apoptosis
Measles
Prolactin signaling pathway
Breast cancer
Biosynthesis of cofactors
Chagas disease
-log10(Pval Adj)
H
Number of genes
2
1
Bacterial invasion of epithelial cells
Synaptic vesicle cycle
Small cell lung cancer
Melanoma
Mucin type O-glycan biosynthesis
Protein digestion and absorption
FoxO signaling pathway
Adherens junction
Endocrine and other factor-regulated calcium reabsorption
Spliceosome
Sphingolipid signaling pathway
PD-L1 expression and PD-1 checkpoint pathway in cancer
-log10(Pval Adj)

Table S6. Potential target genes associated with MASLD pathways using miRWalk database (http://mirwalk.umm.uni-heidelberg.de/, accessed on 12 May 2024), which uses a self-developed algorithm to predict target genes and compares to other publicly available databases (TargetScan, miRDB, miRTarBase).

	**MASLD**	
ADIPOR2	*IKBKB*	*PIK3CD*
AKT2	*IL6R*	*PIK3R1*
AKT3	*IRS1*	*PIK3R3*
BCL2L11	*IRS2*	*PPARA*
CASP7	*ITCH*	*PPARG*
CDC42	*MAPK10*	*PRKAA1*
CEBPA	*MAPK14*	*PRKAA2*
COX6B1	*MAPK8*	*PRKAB2*
COX7B	*MLXIP*	*RAC1*
CXCL8	*NDUFA4*	*RELA*
DDIT3	*NDUFA5*	*RXRA*
ERN1	*NDUFC2-KCTD14*	*SDHC*
FAS	*NDUFS1*	*SDHD*
FASLG	*NR1H3*	*SREBF1*
GSK3B	*PIK3CA*	*UQCRB*
	PIK3CB	*UQCRFS1*

Abbreviations: MASLD, Metabolic dysfunction-associated steatotic liver disease

Table S7. Metabolic pathways associated with MASLD, according to GeneCodis (https://genecodis.genyo.es/, accessed on 12 May 2024) and choosing *Homo sapiens* as a main organism, when taking into account the target genes associated with the disease obtained from miRWalk ((http://mirwalk.umm.uni-heidelberg.de/, accessed on 12 May 2024).

**MASLD**		
• Diabetic cardiomyopathy<br>• Insulin resistance<br>• Alzheimer disease<br>• Chemical carcinogenesis - reactive oxygen species<br>• Lipid and atherosclerosis<br>• FoxO signaling pathway<br>• Neurotrophin signaling pathway<br>• Prion disease<br>• Apoptosis<br>• Insulin signaling pathway<br>• Longevity regulating pathway<br>• Adipocytokine signaling pathway<br>• Alcoholic liver disease<br>• Chagas disease<br>• Yersinia infection<br>• TNF signaling pathway<br>• AMPK signaling pathway<br>• Pancreatic cancer<br>• AGE-RAGE signaling pathway in diabetic complications	• Toll-like receptor signaling pathway<br>• T cell receptor signaling pathway<br>• Longevity regulating pathway - multiple species Fluid shear stress and atherosclerosis<br>• Pathways in cancer<br>• Prolactin signaling pathway<br>• Hepatitis C<br>• Kaposi sarcoma-associated herpesvirus infection<br>• Osteoclast differentiation<br>• Hepatitis B<br>• Pathways of neurodegeneration - multiple diseases<br>• Measles<br>• Autophagy - animal<br>• Colorectal cancer<br>• Human cytomegalovirus infection	• Type II diabetes mellitus<br>• Fc epsilon RI signaling pathway<br>• Growth hormone synthesis; secretion and action<br>• Epstein-Barr virus infection<br>• Shigellosis<br>• PI3K-Akt signaling pathway<br>• C-type lectin receptor signaling pathway<br>• Human immunodeficiency virus 1 infection<br>• B cell receptor signaling pathway<br>• Thermogenesis<br>• VEGF signaling pathway<br>• Sphingolipid signaling pathway<br>• Acute myeloid leukemia<br>• Regulation of lipolysis in adipocytes<br>• EGFR tyrosine kinase inhibitor resistance

Abbreviations: MASLD, Metabolic Dysfunction-Associated Steatotic Liver Disease
